# Female participation or “feminization” of medicine

**DOI:** 10.1007/s10354-022-00961-y

**Published:** 2022-09-02

**Authors:** Verena Steiner-Hofbauer, Henri W. Katz, Julia S. Grundnig, Anita Holzinger

**Affiliations:** Teaching Center, Medical University of Vienna, Spitalgasse 23, 1090 Vienna, Austria

**Keywords:** Feminization, Women in medicine, History of medicine, Gender, Generations

## Abstract

More and more women chose medicine as their profession. Female students and graduates outnumber their male colleagues in Austria and the EU. However, the career paths of men and women differ after a certain point, and more and more female talent is lost along the career stages. Women hold only 30% of professor positions at state medical universities in Austria and only 11.9% of all chief physicians are female. Motherhood and related absence is the main career obstacle, but gender bias and missing role models are also factors hindering women to thrive. Improved working conditions would be beneficial for all members of the medical profession. Future generations (Generation Y, Generation Z) will likely expedite changes toward a better work-life balance and claim the right to find fulfillment besides work. Compatibility of family and work and the chance to individualize career paths could be important factors for employers to find and bind their employees. Additionally, (gender) diverse teams improve the group process and collective intelligence. Therefore, patient care and innovation can only benefit from a diverse medical workforce.

## The history of women in medicine

The history of women in modern academic medicine in Austria started in 1900 when women were permitted to the medical universities in Vienna, Prague, Graz, and Innsbruck. 1903 Margarethe Hönigsberg graduated in medicine at the University of Vienna as the first woman [1]. However, medicine was not always a male-dominated field. In ancient Rome female doctors were practicing in different specialties, Aspasia was one of them; she is pictured in a fresco above the main entrance of the University of Rome next to Socrates, Plato, Archimedes, and Sophocles [2]. In the Middle Ages, monasteries were important centers of medicine. Hundreds of women served as healers and experts in herbal medicine, Hildegard von Bingen was presumably the most famous of them [2]. Women were traditionally involved in health care within the family and the community [3]. Midwifery was—as an example—traditionally in woman’s hands. With the foundation of childbirth clinics, professionalization, academization, and institutionalization of midwifery started. Midwives were cut out and their long-serving, extensive knowledge of childbirth was lost in this process, and male-dominated medical obstetrics was born [4]. Women have been denied the right to formal medical education at universities for many years [3], they were working outside of formal medicine as lay healers and covered medical needs, especially in rural areas, for centuries.

## The number of women in medicine

**Fig. 1:**
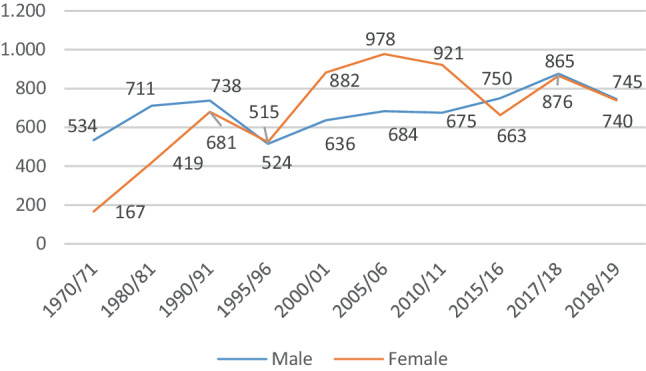
Development of the number of graduations of male and female medical students in Austria, from 1970 to 2019

Since Margarethe Hönigsberg reclaimed a part of medicine, many women followed and chose medicine as their profession. Fig. 1 shows the development of graduations of male and female medical students in Austria since 1970. Since the “baby boomer” generation, born between 1950 and 1964^1^, more and more women studied medicine. The number of female graduates more than doubled from the early seventies to the early eighties. The women of Generation X, born between 1965 and 1979, closed the gap, and since 1995, female students participate in medicine equally or to a greater extent than male students.

**Fig. 2:**
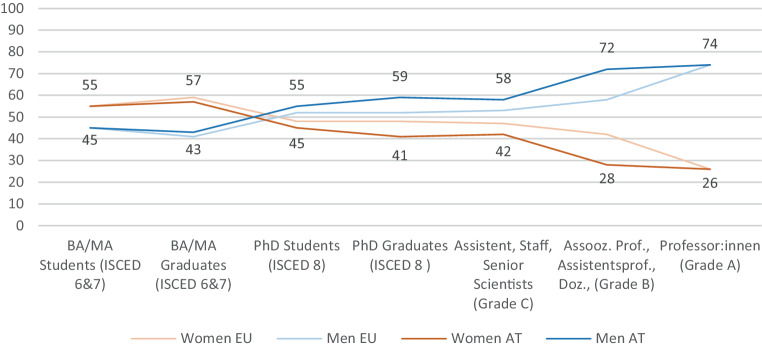
Leaky Pipeline, Proportion of male and female members of different academic career stages within Austria and the EU, in %. [5, 7, 16, 17]. (*ISCED* International Standard Classification of Education)

In Austria 1727 students graduated in medicine in 2019/20, 53.8% of them were women [5] and at the Medical University of Vienna [6] more female medical students (53.7%) were studying medicine in 2020. Furthermore, at the Medical University of Vienna, almost half (47.4%) of the 4059 scientific employees are female, similar to the proportion of female scientific employees at all Austrian Universities (46%). However, after this point, the careers of men and women start to differ substantially. More and more women are dropping out of the carrier path (Fig. 2).

## The “leaky pipeline”

The continuous loss of female talent, the so-called “leaky pipeline”, leads to the fact that at the three state Medical Universities Graz, Innsbruck, and Vienna women held only 30% of all professor positions in 2019/20 [7] and none of them is led by a female rector. However, there are attempts to adjust this imbalance: The university act [8] requires that half of all members of collegiate bodies have to be female [9], and that every university has to enact equality plans and plans to support female careers. In 2020 the Medical University of Vienna awarded eleven of sixteen professor positions to women, due to a special “call for female professors” [10]. This was an extraordinary great amount of women compared to the five years before were only 25% of all new professor positions were offered to female applicants. Even if this might be a start to balance the unequal distribution of high positions—at least in Vienna—the phenomenon of the “leaky pipeline” remains a problem [11]. The decreasing number of women along the academic pathway is a phenomenon seen in Austria and the EU, and in almost all fields of science (Fig. 2; [12]).

Nevertheless, personnel decisions at universities are only one side of this problem. Research is strongly affected too. Female first authorship is associated with female last authorship and female representation on editorial boards of scientific journals. Female researchers are more likely to gain first authorship when the last author is also a woman. The fact that there are few women in high academic ranks leads to the fact that only a few women are last authors of scientific publications. High academic rank and publications are essential preconditions for being constituted on editorial boards and only a few women accomplish the leap through this glass ceiling [11, 13, 14]. Additionally, gender bias is a real phenomenon in the academic world. Moss-Racusin et al. found in a study using fake resumes where only the names were different, that ostensibly male applicants were significantly more likely to be selected for scientific positions than female applicants even if the resumes were completely identical besides the names [15].

**Fig. 3:**
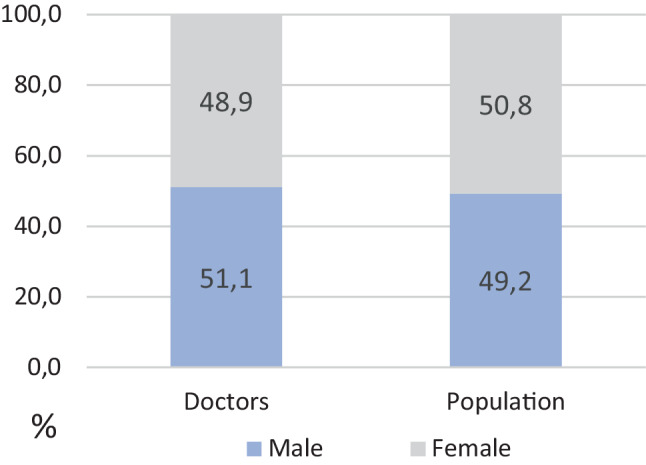
Proportion of male and female population of Austria and proportion of male and female doctors in Austria, in %

However, the leaky pipeline is not solely a phenomenon of the academic world. In 2018, about half of all Austrian doctors were female, almost corresponding to the proportion of women in the Austrian population (50.8%) or the proportion of female citizens within the EU (51.1%) ([18–20]; Fig. 3).

The biggest “surplus” of female doctors can be found in child and adolescent health care: 59.3% of pediatricians and 62% of all child and youth psychiatrists are female. Psychiatry in general is a field with a high percentage of women. In general medicine, the biggest field of medicine, 53.7% of all doctors are female [20]. Female doctors are covering big parts of the basic medical needs like general medicine and working as employed doctors in hospitals [1]. In more prestigious specialties like surgery and also in running a practice, men outnumber women by far [1]. Female doctors lag far behind their male colleagues in all higher positions and their mean income is lower [21]. Only 41% of all consulting physicians and only 11.9% of all chef physicians in Austria are females [1]. Therefore, half of all doctors may be female but the powerful and influential parts of medicine are still dominated by men.

## The term “feminization”

Keeping this data in mind the following question arises: What does “feminization” of medicine mean? As figure three shows women only participate in medicine to the same extent as they are part of the public. Furthermore, “feminization” carries the message that something is changed into a feminine form and that women in medicine are something extraordinary and different from the (masculine) norm. In an equated society there would be no need for a special expression to formalize the equal participation of women [22]. It should be normal that about half of all members of almost every profession are female. Additionally, for many years, there was no need to call the fact that (almost) all doctors were men “masculinity of medicine”[22]. The word “feminization” is particularly problematic because it often carries a negative connotation. The term is used in connection with decreasing prestige of the medical profession [3, 23]. Reputation and salaries are decreasing while the number of female doctors increases. Competing theories exist on how to explain this phenomenon. One hypothesis proposes that the number of female members causes the decline. Others suggest that when a field becomes less prestigious and attractive to men, women are only closing the gap they leave [23, 24]. Additionally, the rising number of female doctors and the theory that they neglect their careers and/or work part-time after childbirth regularly serves as an explanation for the physician shortage [25, 26].

## The inequality of men and women

It should be taken into consideration that for (almost) every woman who starts a family there is a man who is fathering the children [22]. However, fatherhood does not seem to impede a men’s career. Physician shortage should therefore be explained by the socio-cultural phenomenon that childcare and other unpaid care work, like taking care of elderly family members, or kin-keeping are still seen as women’s business. Discriminatory gender stereotypes like this are a real problem and should be reconsidered for the sake of a world where men and women have equal rights and duties. A recent US study showed that during the Covid-19 pandemic 24.6% of female physicians were responsible for child care and schooling compared to only 0.8% of male physicians [27]. At the Medical University of Graz only 8% of all parental leaves are taken by men, at the Medical University of Vienna about 20% [28, 29]. In Vienna, most fathers take parental leaves of only fifty days, whereas mothers take two to four times longer leaves [29]. In Scandinavian/Nordic countries, parental leave is shared equally between mothers and fathers. Additionally, men and women in leading positions, working part-time, are more common [22, 30–32]. Sharing childcare, and the financial losses and risks typically associated with raising children, is a key element of gender equality [33]. A better balance between work and private life could facilitate equal chances not only for women in medicine or other professions but also for men to come to fulfillment in their roles as fathers and/or care-takers. Reducing the total hours of work per week would be a way to facilitate a better balance. To address the concept that women abandon their careers by choice: First and foremost, it has to be considered that leaning against a gender stereotype is a decision not taken lightly. Women in high positions with children are often labeled as bad mothers, abandoning their role as primary care-taker [22]. Additionally, there is a lack of female role models in powerful positions, and specific mentoring for young female professionals [11]. This may lead to the tendency of women to perceive themselves as less capable than men even when they are more capable [34] a fact that was labeled as “confidence gap” [35]. The empowerment of female doctors and scientists is therefore of utmost importance. Networking, mentoring, and career coaching are valuable tools to improve and support the professional development of women [36]. At the Medical University of Vienna the curriculum “schrittweise” and the “Frauen netz.werk Medizin” are installed to address these needs [37]. However, to reduce the risk that programs for the advancement of women help to reproduce stereotypes they must include structural and cultural aspects of organizations as well.

## The future of the medical workforce

Improved working conditions would be beneficial [30] not only regarding family duties but also as a chance to find fulfillment or recovery in a hobby or engagement in other activities besides work. The work of physicians is demanding and doctors often suffer from physical or psychological problems due to the stressful and exhausting working conditions. The incidence of burnout, depression, substance use, and suicidal thoughts is increased among doctors. Long working hours, high workload, and the lack of leisure time to recover are the main reasons for the impaired health of medical professionals [38]. Panagioti et al. [39] state that burnout is a problem of the health care organizations and not of the individual. Impaired fitness to practice and lengthy sick leaves could be averted by increased working conditions and adequate spare-time [40]. Brunet et al. [30] report that working part time not only makes good economic sense it also improves the quality of care. To ensure that high quality medical care can be delivered in the future, health care systems have to face the challenge of a demographic change. The generation “baby boomer”, born between 1950 and 1964, will be in greater need of medical assistance due to their age [41] and at the same time, many doctors of this generation will retire. Members of Generation Y (born between 1980 and 1994), followed by Generation Z (born between 1995 and 2009) [42] will take over. Work-life balance and work-family-reconciliation are more important for male and female members of the Generation Y than for the “baby boomers” [41]. Leisure time is twice as important for Generation Y than for the “baby boomers” [43]. Additionally, the members of Generation Z enter the workforce in the present and the near future. Freedom, health, and family are top-ranked values for Generation Z. Therefore, employers have to prepare for a different kind of target group [44]. A good fit of work and personality, a work with deeper meaning, and a clear separation between work and life that guarantees freedom and enough time to pursue personal goals and happiness are important to male and female members of Generation Y [43] and Generation Z [45, 46]. Gender roles could lose more and more of their binding nature and therefore gender stereotypes and classical role ascriptions may likely fade. It seems possible that biographies of women and men will depend more on individual choices than on traditional gender roles [46]. Soon, the majority of employees will be members of Generations Y and Z and employers will have to meet their requirements to fill their vacancies [47]. This adaption process, for primary care in hospitals or practices, could include providing regular part time employment in hospitals and primary care facilities for all interested persons or a general reduction of the total working hours per week. Enabling of non-bureaucratic splitting and/or sharing of contracts with local health insurance (Kassenverträge). As well as support for the establishment of joint practices [48].

Additionally, it seems important to acknowledge and appreciate the talent and qualities of female doctors and female scientists and recognize the benefits of (not only!) gender-diverse teams. Gender diversity and openness to diversity are predictors of high team performance and gender balance can enhance the group process and improve the level of collective intelligence [49, 50]. A diverse workforce in medicine and research can contribute more to scientific discovery and improved patient care than a homogeneous one could. Therefore all members of this honorable profession should be appreciated as individuals and the discussion at hand could maybe shift from the “feminization” of (former masculine) medicine towards individualization of medicine and medical career paths [51].

## Footnotes

1 Please note that several different definitions exist concerning the start, end and duration of the different “generations”. For this article the definition of Schnetzer, S. (Study: *Junge Deutsche 2019*.) was used.
